# Transcriptome analysis of sugar and acid metabolism in young tomato fruits under high temperature and nitrogen fertilizer influence

**DOI:** 10.3389/fpls.2023.1197553

**Published:** 2023-07-19

**Authors:** Yanjiao Zheng, Zaiqiang Yang, Jing Luo, Yao Zhang, Nan Jiang, Wajid Ali Khattak

**Affiliations:** ^1^Jiangsu Key Laboratory of Agricultural Meteorology, School of Applied Meteorology, Nanjing University of Information Science and Technology, Nanjing, Jiangsu, China; ^2^School of Environment and Safety Engineering, Jiangsu University, Zhenjiang, Jiangsu, China

**Keywords:** transcriptomics, tomato, sugar, organic acid, high temperature, nitrogenous fertilizer

## Abstract

**Introduction:**

Environmental temperature and nitrogen (N) fertilizer are two important factors affecting the sugar and organic acid content of tomato fruit. N is an essential nutrient element for plant growth and development, and plays a key role in regulating plant growth, fruit quality and stress response. However, the comparative effect of different N fertilizer levels on the accumulation of soluble sugar and organic acid in tomato young fruit under high temperature stress and its mechanism are still unknown.

**Methods:**

Three N fertilizer levels (N1, N2, N3) combined with two temperatures (28/18°C, CK; 35/25°C, HT) were used to study the effects of N fertilizer, HT and their interaction on the soluble sugar and organic acid components, content, metabolic enzyme activity and the expression level of key genes in tomato young fruit, revealing how N fertilizer affects the sugar and organic acid metabolism of tomato young fruit under HT at physiological and molecular levels.

**Results:**

The content of soluble sugar and organic acid in tomato young fruit under HT exposure was increased by appropriate N fertilizer (N1) treatment, which was due to the accumulation of glucose, fructose, citric acid and malic acid. High N (N3) and HT exposure had a negative impact on soluble sugar and reduce sugar accumulation. Further studies showed that due to the up-regulation of the expression of sucrose metabolizing enzyme genes (*CWINV2, HK2, SPS, PK*) and sucrose transporter (*SUT1, SUT4, SWEETs*) in tomato, N fertilizer increased the accumulation of soluble sugar by improving the sucrose metabolism, absorption intensity and sucrose transport of fruit under HT exposure. Due to the increase of PEPC gene expression, N fertilizer increased the accumulation of citric acid and malic acid by improving the TCA cycle of fruit under HT exposure.

**Discussion:**

Nitrogen fertilizer can improve the heat tolerance of tomato young fruits by improving sugar metabolism under HT exposure. The results can provide theoretical support for the correct application of N fertilizer to improve the quality of tomato fruit under HT exposure.

## 1 Introduction

Tomato (*Solanum lycopersicum* L.) is one of the most important horticultural crops in the world. The most efficient method for obtaining premium fresh tomatoes for markets is the protective planting of tomato crops (Renau-Morata et al., 2021). China grows protected tomatoes on 810,000 ha cultivated land (Zheng et al., 2022). Since tomato is rich in sugar and organic acid, lycopene, β-carotene, flavonoids, ascorbic acid, phenolic acid, and other nutrients that contributes to the maintenance of human health and people’s taste (Wu et al., 2023), people have higher requirements for tomato flavor and quality. However, in late spring and early summer, the heat generated by solar radiation caused the temperature in the greenhouse to rise sharply. In addition, the greenhouse is a tightly sealed and inadequately ventilated environment, resulting in the interior temperature being 20°C to 30°C higher than the outside temperature (Shamshiri, 2017; Zheng et al., 2020; Zheng et al., 2022). Therefore, high temperature is always a common meteorological disaster in greenhouses.

High temperature (HT) is one of the important environmental factors affecting the sensory and nutritional quality of tomato fruits (Ayenan et al., 2021). Exposure to HT reduces the content of sugar, organic acid, lycopene, and other components in the fruit, thus impacting negatively tomato fruit quality (Hernández et al., 2018; Almeida et al., 2021; Hernández et al., 2022; Mesa et al., 2022). Previous studies mainly focused on the ripening stage of tomatoes; however, little is known about the impact of HT exposure on young fruits. The young fruit stage (< 10 days post-anthesis) is the most sensitive and golden period of the entire fruit development process (Quinet et al., 2019; Zheng et al., 2022). This stage is the peak of cell division activity, which determines the number of cells and, subsequently influences the size, weight, and shape of the fruit (Azzi et al., 2015). In addition to these morphological characteristics, the sensory and nutritional quality characteristics of mature tomato fruits are also identified early, including the accumulation of sugars and organic acids (Azzi et al., 2015; Bauchet et al., 2017). Eighty percent of the sugars in tomato fruits originate from the source organs (leaves) and are transported through the phloem and released into the fruit in the form of sucrose (Powell et al., 2012). Sucrose is broken down into monosaccharide and uridine diphosphate glucose (UDPG) under the action of invertase and sucrose synthase (SS), providing energy to the fruit for the synthesis of starch, cellulose, and various other cellular components (Li et al., 2012). The timely unloading and utilization of sucrose in the sink contributes to the formation of a sucrose concentration difference between the source leaves and the fruit, which promotes the continuous transport of sucrose to the fruit and plays a crucial role in regulating the distribution and intensity of photosynthetic compounds sink (Zhang et al., 2021). Moreover, sucrose and its hexose cleavage products can regulate plant development and stress response through carbon partitioning and sugar signaling (Vijayakumar et al., 2021; Zhang et al., 2022). Several molecular and physiological studies have shown that glucose serves as a signal to promote cell division in embryos during early development (Ruan et al., 2010). Research has shown that heat leads to a reduction in the source/sink ratio, which impairs fruit development by shortening cell division time and reducing cell number and size, ultimately leading to accelerated fruit development and reduced fruit quality (Adams et al., 2001; Bertin, 2005). Therefore, examining the effect of HT on sugar and acid metabolism during the young fruit stage is critical for understanding the effect of HT on fruit quality of tomato.

In addition to HT, nitrogen (N) fertilizer application during cultivation can also affect fruit development and quality (Erdinc et al., 2018). Experimental results indicate that sugar and organic acid content in tomato fruits is proportional to N fertilizer at a certain level, which improves fruit quality (Hernández et al., 2020; Ronga et al., 2020). Although N has a positive effect on fruit quality, excess N affects the carbon (C) and N balance, decreasing the amount of soluble sugar in tomato fruit, while increasing the organic acid content, and decreasing fruit flavor quality (Bénard et al., 2009; Gruda et al., 2018; Truffault et al., 2019).

Considering that both HT and N fertilizers are important factors affecting fruit quality, some studies have investigated the comprehensive effects of HT and N. N can reduce the reactive oxygen species toxicity in rice (Wei et al., 2018) and corn (Yan et al., 2017) to resist HT by increasing antioxidant concentration and photosynthetic utilization rate. Xu et al. (2022) found that adequate N fertilization can mitigate the adverse effects of HT during grain filling on milling quality and chalky occurrence to some extent. Previous research has shown that N fertilizer increases the sensitivity of maize (Ordóñez et al., 2015), rice (Tang et al., 2019), and wheat (Elía et al., 2018) to HT, and these studies suggest that excessive N exacerbates yield losses due to temperature increase. These results highlight the complex relationship between N levels and HT, suggesting that each crop species may have the best combination of HT and N levels (Yang et al., 2015). Unfortunately, few studies have shown the possibility of such interactions between HT and N fertilizer rates in tomato young stage.

The development of high-throughput sequencing and genome sequencing data for tomato offers important methods and references for exploring complex regulatory networks and identifying new key genes (Su et al., 2022). With the improved of RNA sequencing (RNA-seq) efficiency, transcriptome analysis has identified many genes involved in tomato response to heat stress. For example, Gul et al. (2021) and Su et al. (2022) discovered key genes associated with heat tolerance in tomato using RNA-seq technology. In addition, the key genes involved in the biochemical pathway and expression characteristics of transport, accumulation, and metabolism of sugars and organic acids in tomato fruit were also discovered using RNA-seq technology (Li et al., 2022; Tao et al., 2022; Wu et al., 2023). More importantly, the key genes that respond to N and regulate the metabolism of sugar and organic acids in fruits have been discovered (Wang et al., 2021; Cao et al., 2022). With the advancement of molecular biology technology, more genes involved in plant response to abiotic stress will be discovered and identified in the future.

We hypothesize that there is an interaction between HT and N fertilizer in young tomato fruit, and we also hypothesize that N fertilizer resists heat stress by regulating the activity of sugar and organic acid metabolism enzymes and gene expression. In this paper, three N levels and two temperature treatments were applied to compare the biochemical pathways of sugar and acid metabolism in tomato fruits under different N and temperature treatments. The objective of this study was to determine the interaction between N and HT and to investigate their comprehensive effects on the content, composition, and metabolic enzyme activity of soluble sugars and organic acids in young tomato fruits. RNA Seq analysis was used to identify the key genes involved in sugar and organic acid metabolism in tomato young fruits exposed to N and HT. The research results may provide a theoretical basis for appropriate N application rates under HT conditions to improve heat resistance in young tomato fruits.

## 2 Materials and methods

### 2.1 Experimental design

The experiment was conducted at the Agricultural Meteorological Research Station of Nanjing University of Information Science and Technology (32°13´N, 118°43´E; 29 m above mean sea level) in Nanjing, Jiangsu Province, China. “Powder crown” was used as test material, and once it formed 4-5 true leaves, it was planted in a flower pot with dimensions of 28 cm (height) × 34 cm (upper caliber) × 28 cm (bottom diameter), dated February 27, 2022. Soils contained fertilizers, including organic carbon (12.93 g kg^-1^), organic matter (22.29 g kg^-1^), available phosphorus (69.36 mg kg^-1^), available potassium (13.8 mg L^-1^), and total nitrogen (0.13%). Before transplanting, phosphate and potassium fertilizer were applied to the planting soil all at once as base fertilizer, with the amount of phosphate fertilizer (monocalcium phosphate) being 200 kg ha^-1^ (5.67 g plant^-1^) and the amount of potassium fertilizer (potassium sulphate) being 300 kg ha^-1^ (8.51 g plant^-1^). N fertilizer was applied in three levels: 188 kg ha^-1^ (N1), 250 kg ha^-1^ (N2), and 313 kg ha^-1^ (N3). The N application rate of 250 kg ha^-1^ is the rate currently used by the local farmers. N fertilizer (urea) was used as a topdressing (hole fertilization). Based on the planting density of tomatoes, we calculated that the N application for each tomato plant during the entire growth period was N1 (5.33 g plant^-1^), N2 (7.10 g plant^-1^), and N3 (8.88 g plant^-1^), respectively. We applied N in a ratio of 3:3:2:2 during the seedling, flowering, second, and fourth ear fruit expansion stages of tomato plants. Since our research focused on tomato young fruits, we didn’t consider topdressing after flowering. The specific N application was shown in **Table 1**. Each N level treatment was repeated 20 times (i.e. 20 pots), with one tomato seedling per pot, resulting in a total of 60 pots of tomatoes. After 10 days of transplanting (March 9, 2022), tomato seedlings with consistent growth were randomly selected for the first fertilization as topdressing during the seedling stage. Transplanted tomato potted plants grew in a Velon glass greenhouse. The greenhouse structure has an area of 30 m in length × 9.6 m in width, and 4.5 m in height with north-south orientation. The distance between pots was 35 × 40 cm, and watered every 2 days according to the needs of plant growth to maintain appropriate growth conditions. Until the first order of tomato flowers fully bloomed, N was applied again to the corresponding N level pots as topdressing (April 4, 2022). After 5 days, we conducted a heat stress test, during which the fruits that successfully set were marked and the date of set was recorded. To evaluate the interaction between temperature and nitrogen fertilizer application, we randomly divided pot plants with the same N level into two groups and simultaneously treated them at two different temperatures for 5 days (April 9, 2022)Two artificial climate chambers (Convion BDW40, Canada) were used, one designed for suitable temperature (CK) and the other designed for heat stress (HT). The temperature range of CK treatment was 18-28°C (with an error of ± 0.5°C), and the daily average temperature was 22.7°C; the temperature range of HT treatment was 25-35°C (with an error of ± 0.5°C), the daily average temperature was 29.6°C. The daily variation of temperature was similar to the daily variation of temperature in the natural atmosphere of Nanjing. The minimum and maximum temperatures occurred at 5:00 and 14:00 respectively, as shown in **Figure S1**. The tomato plants in the artificial climate chamber were arranged in three rows, one row (10 pots) at each N level. Plants at the same N level were randomized. The two artificial climate chambers had the same climate conditions except for temperature treatment. Photosynthetically active radiation (PAR) of the artificial climate room was set as follows: 6:00-10:00 (800 μmol m^-2^ s^-1^), 11:00-14:00 (1000 μmol m^-2^ s^-1^), 15:00-18:00 (800 μmol m^-2^ s^-1^), and the remaining period was 0 μmol m^-2^ s^-1^. The humidity was set to 60% at 6:00-18:00 in the daytime, 80% at night, and the CO_2_ concentration was set to 400 μmol mol^-1^. During heat stress, plants were watered once a day to ensure the necessary water for normal plant growth. Six treatments were set: N1 application in the CK environment (CKN1); N2 application in the CK environment (CKN2); N3 application in the CK environment (CKN3); N1 application in the HT environment (HTN1); N2 application in the HT environment (HTN2); N3 application in the HT environment (HTN3). After the heat stress test, three tomato young fruit samples that set on the same day were taken from each treatment, the skin and seeds were removed, the flesh was cut into silk and placed in a zipper bag. Then, it was quickly frozen in liquid nitrogen and stored at -80°C until subsequent analysis.

**Table 1:** Application period and amount of N fertilizer (urea) (g plant^-1^).

Growth stage	First application of N fertilizer	Second application of N fertilizer
	Seedling stage (10 days after transplanting, March 9, 2022)	Flowering period (the first order flower is in full bloom, April 4, 2022)
N1	1.60	1.60
N2	2.13	2.13
N3	2.66	2.66

### 2.2 Measurements and methods

#### 2.2.1 Determination of soluble sugars

Fructose, glucose, and sucrose contents were measured following the procedure given by Ma et al. (2019) with slight modifications. The soluble sugars in the fruit were determined using the colorimetric method. The samples of tomato (5 g) were finely grounded using mortar and pestle with the addition of 10 ml of 80% ethanol, stirred evenly and keep it in a water bath at 80-85°C for 40 min. After cooling, added 0.2 g activated carbon for decolorization for 20 minutes, then centrifuged at 4000 r min^-1^ for 30 minutes. Take the supernatant into a 25 mL volumetric flask. This extraction procedure was repeated three times and the supernatants were combined and finally dilute to 25 mL with distilled water. The solution was then used for the sugar analysis.

#### 2.2.2 Determination of organic acids

The contents of organic acids were measured by high performance liquid chromatography (HPLC) using the method reported by Zheng et al. (2022) with some modifications. The samples of tomato (1 g) were finely grounded using mortar and pestle with the addition of 3 mL of distilled water. The solution was transferred into a centrifuge tube and added distilled water to make a constant volume of 5 mL, and then centrifuged at 4000 r min^-1^ for 10 minutes at 4°C. For each sample, 20 μL of the supernatant was filtered through a 0.45 μm membrane and then injected into the HPLC system for analysis. 0.01 mol L^-1^ KH_2_PO_4_ (pH 2.55): methanol (97/3) (v/v) was used as the mobile phase with a flow rate of 0.5 mL min^-1^. The column was 0.5 µm C18 column (250 mm×4.6 mm, Agilent, USA) at 30°C. Organic acids were detected at a wavelength of 210 nm.

#### 2.2.3 Extraction and determination of sucrose and organic acids metabolism-related enzymes

Enzymes involved in sucrose metabolism were extracted following the procedure proposed by Wu et al. (2020) with slight modifications. All procedures were conducted at 4°C. Tomato samples (1 g) were homogenized with 10 mL of 100 mmol L^-1^ phosphate buffer (pH 7.5) containing 10 mmol L^-1^ MgCl_2_, 1 mmol L^-1^ EDTA, 0.1% (v/v) tritonX-100, 0.2% 2-Hydroxy-1-ethanethiol, and 2% polyvinyl pyrrolidone (PVP). The homogenate was centrifuged at 4000 r min^-1^ for 15 min. The supernatant (crude enzyme extract) was used to determine the activity of enzymes involved in sucrose metabolism (acid invertase (AI), neutral invertase (NI), sucrose synthase-cleavage (SSc), sucrose synthase-synthesis (SSs), sucrose phosphate synthase (SPS)) following the procedure given by Zheng et al. (2022) with slight modifications.

Enzymes involved in organic acids metabolism were extracted following the procedure proposed by (Wu et al., 2020) with slight modifications. Tomato samples (10 g) were homogenized with 10 mL of 200 mmol L^-1^ Tris-HCl buffer solution (pH 8.2) containing 600 mmol L^-1^ sucrose and 10 mmol L^-1^ isoascorbic acid. The homogenate was centrifuged at 4000 r min^-1^ for 20 min, and then taken the supernatant and made up to the final volume (10 mL) with ice-cold extract buffer as crude enzyme solution. The solution was used to determine the activity of enzymes involved in organic acids metabolism, which included phosphoenolpyruvate carboxylase (PEPC), malate dehydrogenase (MDH), malic enzyme (ME), citrate synthase (CS), cytosolic aconitase (cyt-ACO), mitochondrion aconitase (mit-ACO) and isocitrate dehydrogenase (IDH).

#### 2.2.4 Transcriptome sequencing analysis

The transcriptome analysis of tomato pulp grown under two temperatures and three N levels was carried out. There were 3 biological replicates per sample, a total of eighteen samples. Total RNA was extracted from the tissue using the TrizolLysis Reagent reagent (Qiagen, Germany) according to the manufacturer’s instructions. garose gel electrophoresis Biowest Agarose (Biowest, Spain) was used to detect whether RNA was degraded, and Nanodrop 2000 (Thermo Fisher Scientific, USA) was used to detect the concentration and purity of the extracted RNA. Then RNA quality was determined by 5300 Bioanalyser (Agilent, USA) and quantified using the ND-2000 (NanoDrop Technologies, USA). Approximately, 1μg of RNA per sample was used for cDNA library construction by Illumina NovaSeq Reagent Kit for Illumina (New England BioLabs, USA) following the manufacturer’s recommendations. The NovaSeq 6000 sequencing platform was used for high-throughput sequencing, and 150 bp double ended data was obtained. The sequencing process was completed by Shanghai Majorbio. To ensure the accuracy of subsequent biological information analysis, the original sequencing data was filtered through fastp (Renau-Morata et al., 2021) software to obtain high-quality clean data. The original data after quality control was compared with the reference genome (ftp://ftp.solgenomics.net/genomes/Solanum_lycopersicum/Heinz1706/assembly/build_4.00/) through HiSat2 software to obtain mapped data (reads), and the quality of the transcriptome sequencing comparison results was evaluated. RSEM was used to quantify gene abundances. Essentially, differential expression analysis was performed using the DESeq2 (Liao et al., 2022). DEGs with |log2FC| > 2 and FDR ≤ 0.05 (DESeq2) were considered significantly different expressed genes. In addition, functional-enrichment analysis including GO and KEGG was performed to identify which DEGs were significantly enriched in GO terms and metabolic pathways at BH-corrected P-adjust ≤ 0.5 compared with the whole-transcriptome background (Li et al., 2021).

### 2.3 Statistical analysis

Data were analyzed and graphed (bar graphs and heat maps) using Origin 2023 (Electronic Arts Inc, USA). The data significance was tested using the LSD method in one-way ANOVA with a significance level of *P* < 0.05. Values were presented as the means standard error (SE) in at least biological triplicate for each measurement.

## 3 Results

### 3.1 Effect of nitrogen-temperature treatments on sucrose metabolism

**Figure 1:**
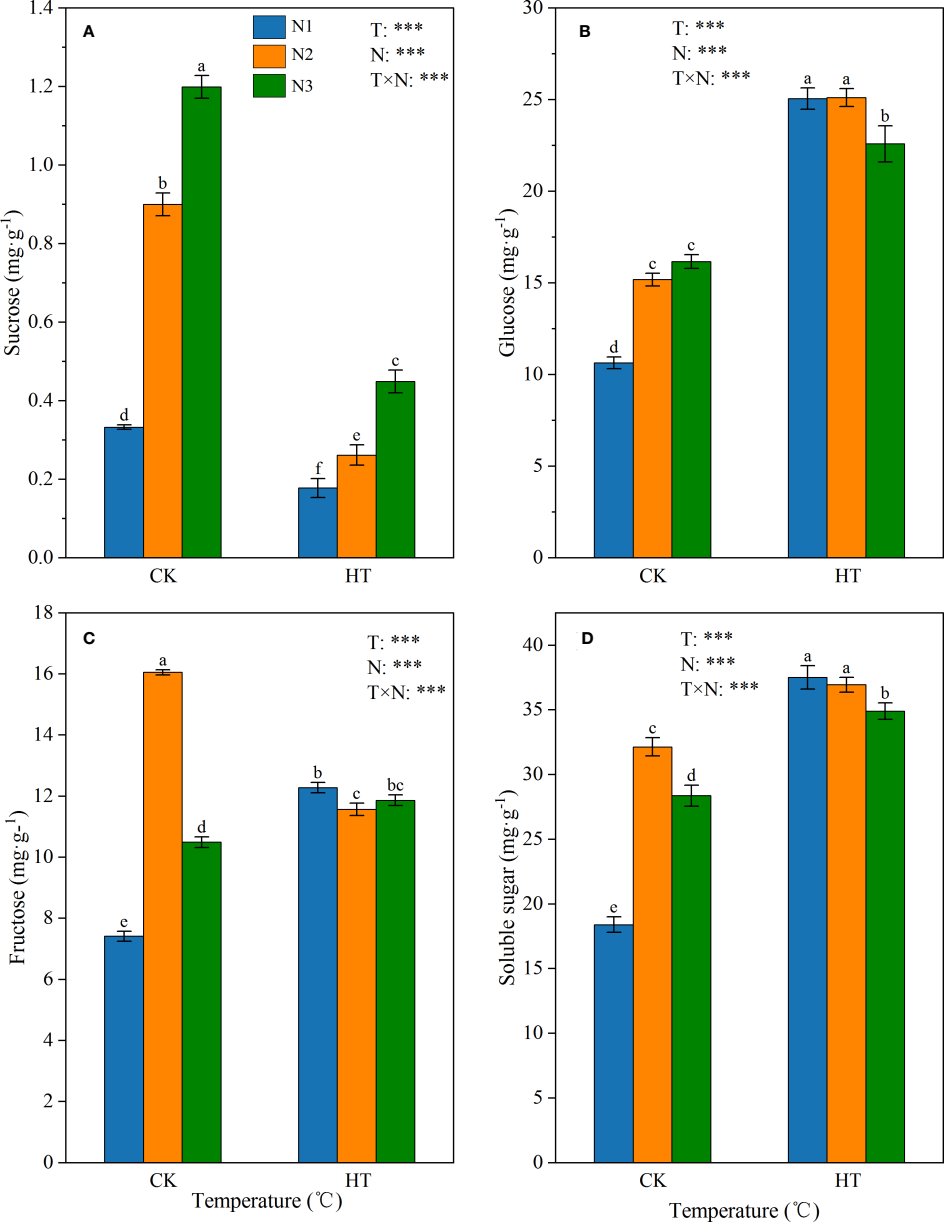
The contents of sucrose **(A)**, glucose **(B)**, fructose **(C)**, and soluble sugar **(D)** in tomato young fruits under different nitrogen (N1, N2, and N3) and temperature (CK, control; HT, high temperature) treatments. Different alphabets (a, b, and c) indicate significant differences between means at *P* < 0.05 using the LSD method. Error bars indicate standard errors (n=3); T, N, and T × N represent temperature treatment, N fertilizer treatment, and their interaction, respectively; *** represents the significant level at P < 0.001.

Under different temperatures (CK and HT), the main sugars (i.e. sucrose, fructose, and glucose) involved in sucrose metabolism in tomato young fruits showed varying degrees of response to N levels (**Figure 1**). The results showed that temperature, N levels, and their interactions had a significant impact on sucrose, glucose, and fructose content (*P* < 0.001). Under the same N level, HT exposure significantly reduced sucrose content. In addition, under the same temperature environment (CK and HT), the sucrose content was ranked as N3>N2>N1 (**Figure 1A**). Contrary to sucrose, HT exposure significantly increased glucose content at the same N level. In the CK environment, the glucose content increased with the increase of N application, while in the HT environment, N3 was significantly lower than N2 (10.045%) (**Figure 1B**). For fructose content, at the N1 and N3 levels, compared to CK, HT exposure significantly increased fructose content by 65.646% and 13.049%, respectively (**Figure 1C**). The highest soluble sugar content was observed under HTN1 treatment (**Figure 1D**).

**Figure 2:**
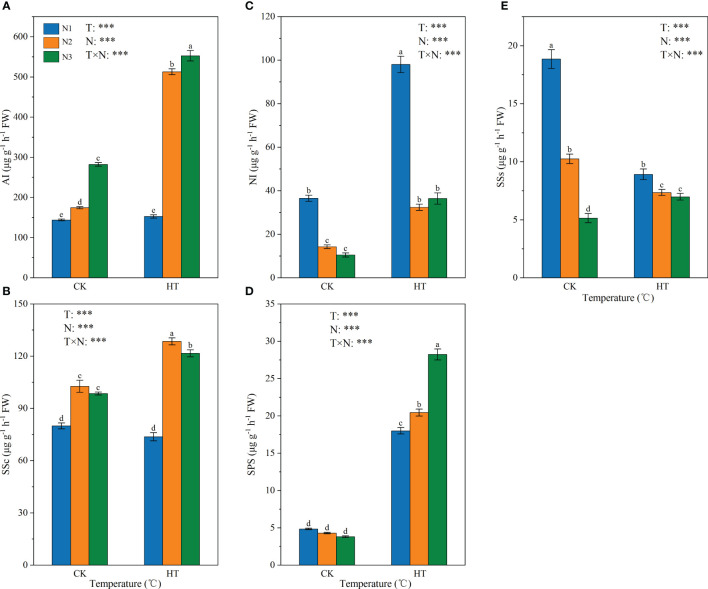
The activity of AI **(A)**, SSc **(B)**, NI **(C)**, SPS **(D)**, and SSs **(E)** in tomato young fruits under different nitrogen (N1, N2, and N3) and temperature (CK, control; HT, high temperature) treatments. AI, acid invertase; NI, neutral invertase; SSc, sucrose synthase-cleavage; SSs, sucrose synthase-synthesis; SPS, sucrose phosphate synthase. Different alphabets (a, b, and c) indicate significant differences between means at *P* < 0.05 using the LSD method. Error bars indicate standard errors (n=3); T, N, and T × N represent temperature treatment, N fertilizer treatment, and their interaction, respectively; *** represents the significant level at P < 0.001.

**Figure 2** shows the effect of N fertilizer on the key enzyme activity of sucrose metabolism (i.e. AI, NI, SSs, SSc, and SPS) in tomato young fruits under different temperature environments. The results showed that temperature, N levels, and their interactions had a significant impact on AI, NI, SSs, SSc, and SPS enzyme activities (*P* < 0.001). Fertilizing to N2 and N3, HT exposure increased AI activity by 193.647% and 95.716%, respectively (**Figure 2A**), and increased SSc activity by 25.177% and 23.416%, respectively (**Figure 2B**). At three N levels, HT exposure significantly increased the activity of NI and SPS (**Figures 2C, D**); on the contrary, HT exposure at the N1 and N2 reduced SSs activity by 52.673% and 26.223%, respectively. In addition, under the same temperature, the activity of SSs was negatively correlated with N application (**Figure 2E**).

### 3.2 Effect of nitrogen-temperature treatments on organic acid metabolism

**Figure 3:**
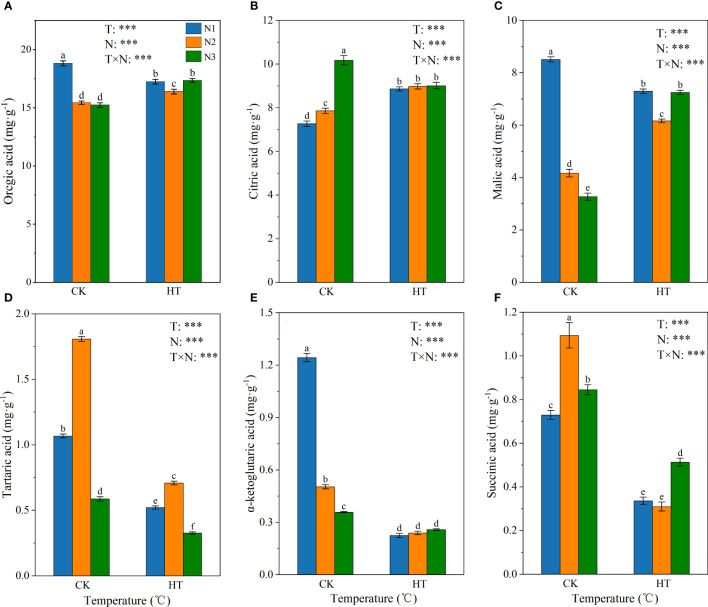
The content of organic acid **(A)**, citric acid **(B)**, malic acid **(C)**, tartaric acid **(D)**, α-ketoglutaric acid **(E)** and succinic acid **(F)** in tomato young fruits under different nitrogen (N1, N2, and N3) and temperature (CK, control; HT, high temperature) treatments. Different alphabets (a, b, and c) indicate significant differences between means at *P* < 0.05 using the LSD method. Error bars indicate standard errors (n=3); T, N, and T × N represent temperature treatment, N fertilizer treatment, and their interaction, respectively; *** represents the significant level at P < 0.001.

**Figure 3** shows the effect of N fertilizer on organic acid composition (i.e. citric acid, malic acid, tartaric acid, α-ketoglutaric acid, and succinic acid) at different temperatures. The variance results showed that temperature, N levels, and their interactions had a significant impact on the organic acid compositions (*P* < 0.001). Under N1 and N2 treatments, compared to CK, HT exposure increased the citric acid content by 21.975% and 14.284%, respectively, while under N3 treatment, HT exposure reduced the citric acid content by 11.462% (**Figure 3B**). Fertilizing to N2 and N3, HT exposure increased the malic acid content by 47.743% and 121.448%, respectively (**Figure 3C**). On the contrary, at the same N level, HT exposure significantly reduced the content of tartaric acid, α-ketoglutaric acid, and succinic acid, compared to CK (**Figures 3D–F**). Overall, the organic acid content was highest at CKN1, and fertilizing to N2 and N3, HT exposure increased the organic acid content by 6.253% and 13.894%, respectively. In addition, CKN2 and CKN3, HTN1 and HTN3 treatments had no significant effect on organic acid content (**Figure 3A**).

**Figure 4:**
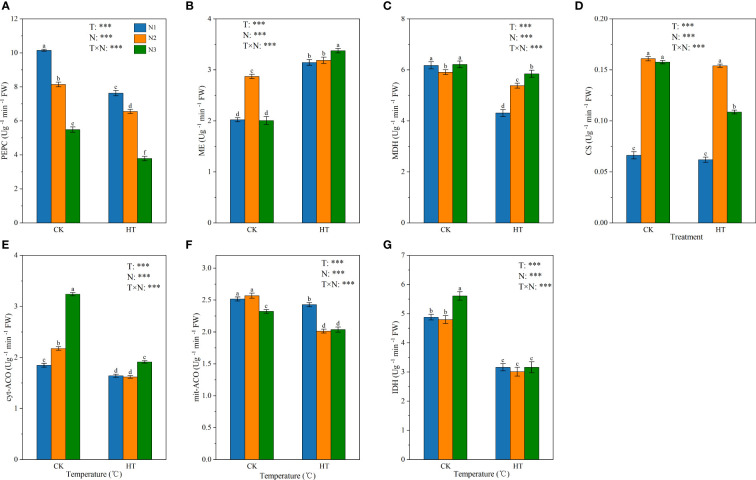
The activity of three malate metabolism-related enzymes (PEPC **(A)**, ME **(B)**, and MDH **(C)**) and four citric acid metabolism-related enzymes (CS **(D)**, cyt-ACO **(E)**, mit-ACO **(F)**, and IDH **(G)**) in tomato young fruits under different nitrogen (N1, N2, and N3) and temperature (CK, control; HT, high temperature) treatments. PEPC, phosphoenolpyruvate carboxylase; MDH, malate dehydrogenase; ME, malic enzyme; CS, citrate synthase; cyt-ACO, cytosolic aconitase; mit-ACO, mitochondrion aconitase; IDH, isocitrate dehydrogenase. Different alphabets (a, b, and c) indicate significant differences between means at *P* < 0.05 using the LSD method. Error bars indicate standard errors (n=3); T, N, and T × N represent temperature treatment, N fertilizer treatment, and their interaction, respectively; *** represents the significant level at P < 0.001.

**Figure 4** shows the effect of N fertilizer on the key enzyme activity of organic acid metabolism (i.e. PEPC, ME, MDH, CS, cyt-ACO, mit-ACO, and IDH) in tomato young fruits under different temperature environments. The results showed that temperature, N levels, and their interactions had a significant impact on the key enzyme activities (*P* < 0.001). At the same N level, compared to CK, HT exposure significantly reduced the enzyme activities of PEPC, MDH, cyt-ACO, mit-ACO, and IDH (**Figures 4A, C, E–G**). At HT environment, PEPC enzyme activity decreased with the increase of N application, while MDH enzyme activity increased with the increase of N application, and IDH enzyme activity had no significant effect on N application. At the N3 level, HT exposure significantly reduced CS enzyme activity by 31.078%, while at the N1 and N2 levels, CK and HT temperature treatments had no significant effect on CS enzyme activity (**Figure 4D**).

### 3.3 Quality assessment of transcriptome sequencing results

To further explore the molecular events of sugar and organic acid metabolism as affected by N and temperature, RNA sequence analysis was performed on 18 tomato young fruit samples from 6 treatments and 3 biological replicates. The quality assessment of transcriptome sequencing was shown in **Table 2**. At least 46719846 raw reads were obtained in all samples, after screening and filtering, 138,086,648 clean reads and 44977486 clean bases were obtained. The proportion of clean reads in each sample was greater than 96%, Q20 was more than 98%, Q30 was more than 94%, and the GC content was higher than 43%. These results indicated that the sequencing quality was favorable for subsequent analyses.

**Table 2:** Statistical results of transcriptome sequencing of young tomato fruit.

Sample	Raw reads	Clean reads	Clean bases	Q20 (%)	Q30 (%)	GC content (%)
CKN3-1	59711860	57773462	8389310670	98.09	94.41	43.56
CKN3-2	55895760	54011280	7945575293	98.14	94.50	43.45
CKN3-3	50871570	49138204	7212091624	98.12	94.52	43.18
CKN2-1	48281446	46532680	6894668437	98.12	94.47	43.59
CKN2-2	54235798	52550584	7745223564	98.10	94.44	43.50
CKN2-3	58843248	56702642	8324739785	98.16	94.60	43.72
CKN1-1	46859574	44977486	6626960613	98.00	94.15	43.18
CKN1-2	46719846	45752190	6726803489	98.44	95.11	43.38
CKN1-3	56527124	54751366	8076012865	98.22	94.74	43.30
HTN3-1	60819686	58929074	8628629685	98.19	94.67	43.43
HTN3-2	53312832	51365932	7550787419	98.07	94.38	43.39
HTN3-3	52782316	50938414	7415134572	98.19	94.74	43.49
HTN2-1	58130288	56421662	8110418149	98.12	94.49	43.59
HTN2-2	49348802	47686502	6967694734	98.21	94.72	43.62
HTN2-3	55090132	53376982	7762799158	98.17	94.65	43.61
HTN1-1	50823150	49075548	7247235508	98.16	94.64	43.52
HTN1-2	51939976	50196488	7375184416	98.07	94.35	43.49
HTN1-3	52178858	50151088	7411129052	98.05	94.34	43.44
mean	53465125.89	51685088				
total	962372266	930331584				

Q20: The percentage of the number of bases with a Qphred value not less than 20 in the total number of bases. Q30: The percentage of the number of bases with a Qphred value not less than 30 in the total number of bases. GC Content: The percentage of the sum of the quantities of G and C in the total number of bases in high-quality reads.

### 3.4 Screening results of DEGs

**Figure 5:**
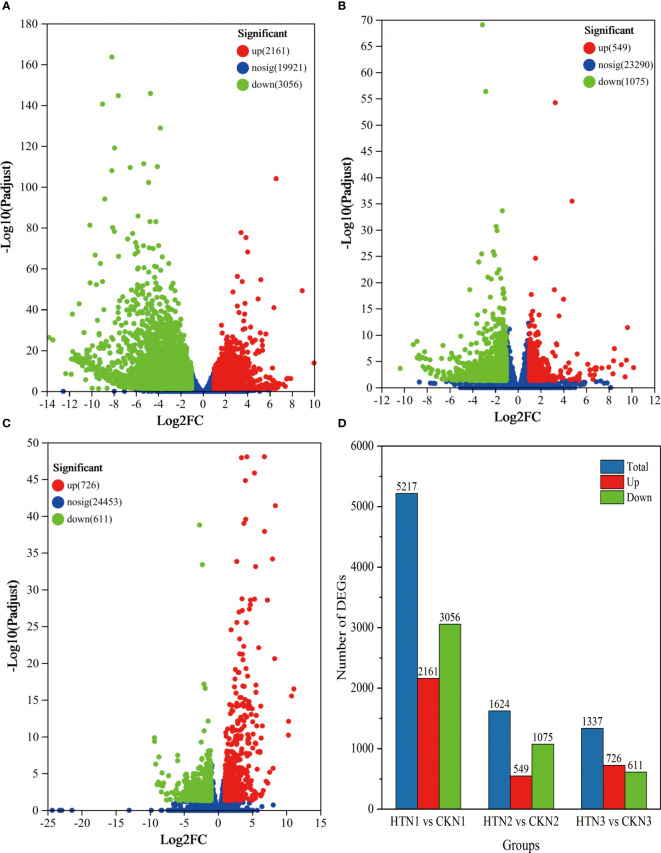
The volcano plot of DEGs. DEGs were selected by |log 2 FC| > 2 and padj < 0.05. The x-axis shows the fold change in gene expression, and the y-axis shows the statistical significance of the differences. **(A–C)** represent the GO functions of DEGs at HTN1 vs CKN1, HTN2 vs CKN2, and HTN3 vs CKN3. **(D)** The bar plot of DEG number generated by comparison of HTN1 vs CKN1, HTN2 vs CKN2, and HTN3 vs CKN3.

To study the transcription of tomato fruit under HT at the same N level, we compared and analyzed the transcriptome data of HTN1 vs CKN1, HTN2 vs CKN2, HTN3 vs CKN3. According to the volcano map **Figure 5**, a total of 5217 DEGs were screened from HTN1 vs CKN1. Among them, 2161 genes were up-regulated and 3056 genes were down-regulated in the HTN1group compared with the CKN1 group, respectively (**Figure 5A**). In HTN2 vs CKN2 group, a total of 1624 DEGs were screened, including 549 up-regulated genes and 1075 down-regulated genes, respectively (**Figure 5B**). A total of 1337 DEGs were screened out in HTN3 vs CKN3 group, of which 726 genes were up-regulated and 611 genes were down-regulated, respectively (**Figure 5C**). The group of HTN1 vs CKN1 had the largest value of DEGs, and the number of DEGs in the HTN2 vs CKN2 groups was greater than that in HTN3 vs CKN3 groups (**Figure 5D** and **Table S1**), indicating that some genes are differentially expressed in the various treatments of nitrogen-temperature and that the number of differentially expressed genes decreases with the increase of N fertilizer application.

### 3.5 GO functional categorization of DEGs

**Figure 6:**
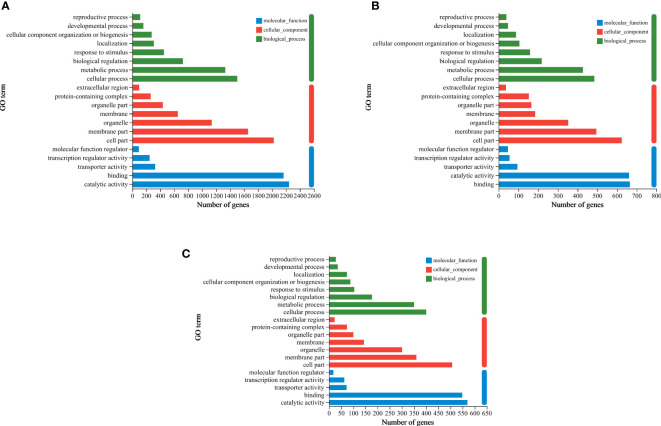
GO enrichment column plot. Differentially expressed genes generated by a comparison of **(A)** HTN1 vs CKN1, **(B)** HTN2 vs CKN2, **(C)** HTN3 vs CKN3 are grouped into different GO terms of three ontologies: biological process, cellular component and molecular function.

To further characterize nitrogen-temperature treatment-responsive DEGs, GO analysis was performed. The top-20 GO enrichment histograms of the DEGs was presented in **Figure 6** and **Table S2**. According to GO functional analysis, DEGs were mainly divided into three functional categories: molecular function (MF), cellular component (CC), and biological process (BP). The three groups (HTN1 vs CKN1, HTN2 vs CKN2, and HTN3 vs CKN3) of generated DEGs showed certain similarity in GO enrichment analysis. There were eight items enriched in BP, among which GO: 0009987 (cellular process), GO: 0008152 (metabolic process), and GO: 0065007 (biological regulation) were enriched in more DEGs. There were seven entries enriched in CC, among which GO: 0044464 (cell part), GO: 0044425 (membrane part), and GO: 0043226 (organelle) were enriched in more DEGs. There were five entries enriched in MF, among which GO: 0003824 (catalytic activity) and GO: 0005488 (binding) were enriched in more DEGs. The functional classification results of DEGs in metabolic process, cell part and catalytic activity showed that the metabolism and enzyme catalytic function of tomato young fruits would change with the variation of temperature and N fertilizer application.

### 3.6 KEGG enrichment analysis of DEGs

**Figure 7:**
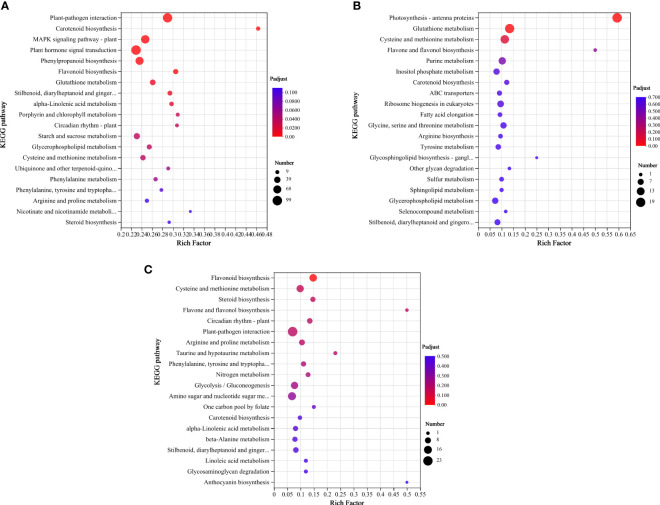
KEGG enrichment bubble plot. Differentially expressed genes generated by a comparison of **(A)** HTN1 vs CKN1, **(B)** HTN2 vs CKN2, **(C)** HTN3 vs CKN3. Rich factor represents the ratio of the number of DEGs enriched by the pathway to the number of annotated genes. The color bar represents the significance test p value adjusted for multiple hypothesis testing. The number represents the number of DEGs enriched in the pathway.

To further investigate the possible molecular functions of DEGs in HTN1 vs CKN1, HTN2 vs CKN2, and HTN3 vs CKN3, KEGG enrichment analysis was performed, and 20 pathways with the most significant enrichment were selected for representation a bubble chart (**Figure 7** and **Table S3**). The three groups were enriched with 1704, 549, and 461 DEGs, respectively, in which the categories of biosynthesis, signal transduction, and metabolism were the most enriched pathways. Notably, in the metabolism category, the most enriched pathway was carbohydrate metabolism, amino acid metabolism, and lipid metabolism. KEGG analysis showed that many DEGs were involved in starch and sucrose metabolism, purine metabolism, glycolysis/gluconeogenesis, glyoxylate and dicarboxylate metabolism, fructose and mannose metabolism, amino sugar and nucleotide sugar metabolism, and glycosaminoglycan degradation. These pathways were related to sugar and acid metabolism in tomato young fruits, indicating that nitrogen-temperature treatments during this period may have a significant regulatory effect on sugar and organic acid metabolism.

### 3.7 Screening and differential expression of sucrose and organic acid-related genes

To further analyze the DEGs encoding enzymes related to sucrose and organic acid metabolism in HTN1 vs CKN1, HTN2 vs CKN2, and HTN3 vs CKN3, 115 DEGs related to sucrose and organic acid metabolism were screened from RNA-seqdata. Among them, 74 DEGs related to sucrose metabolism were compared in three groups, including 31 down-regulated genes and 26 up-regulated genes in every group. There were 41 organic acid-related DEGs in the three groups, including 21 down-regulated genes and 20 up-regulated genes in HTN1 vs CKN1 group, and 19 down-regulated genes and 22 up-regulated genes in HTN2 vs CKN2 and HTN3 vs CKN3 groups. In the three groups, the genes related to sucrose and organic acid metabolism were differentially expressed in different N-temperature treatments.

**Figure 8:**
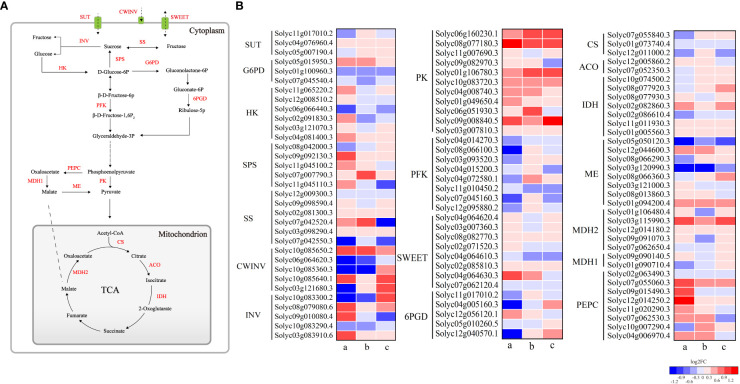
**(A, B)** represent sugar and organic acid metabolic pathways and heatmaps of gene expression for related enzymes, respectively. Small letters a, b and c represent HTN1 vs CKN1, HTN2 vs CKN2 and HTN3 vs CKN3, respectively. SUT, sucrose transporter; SWEET, sugars will eventually be exported transporters; INV, invertase; CWINV, cell wall invertase; SS, sucrose synthase; SPS, sucrose phosphate synthase; HK, hexokinase; G6PD, glucose 6-phosphate dehydrogenase; 6PGD, 6-phosphogluconate dehydrogenase; PFK, phosphofructokinase; PK, pyruvate kinase; PEPC, phosphoenolpyruvate carboxylase; MDH, malate dehydrogenase; ME, malic enzyme; CS, citrate synthase; ACO, aconitase; IDH, isocitrate dehydrogenase.

In the process of sugar metabolism, the DEGs were mainly annotated in the metabolisms of sucrose, fructose, and glucose (**Figure 8A**). Sucrose produced by photosynthesis is mainly converted into hexose by invertase. After HT exposure, the expression of *Invertase* was changed with the log2FC ranging from -4.483 to 3.545 (**Figure 8B**). HTN1 and HTN2 decreased the expression level of invertase and inhibited the conversion of sucrose into fructose and glucose. Additionally, six genes related to *SS* were found in 57 sucrose-related genes, of which HTN1 vs CKN1 and HTN3 vs CKN3 were down-regulated and HTN2 vs CKN2 were up-regulated. *SPS* had five DEGs, of which most were up-regulated at HT exposure. The expression of *hexokinase* (*HK*) increased at HTN1 vs CKN1, while it decreased at HTN2 vs CKN2 and HTN3 vs CKN3. *Glucose 6-phosphate dehydrogenase* (*G6PD*) had three DEGs, two of which were down-regulated. *6-phosphogluconate dehydrogenase* (*6PGD*) and *phosphofructokinase* (*PFK*) had four and eight DEGs, respectively, of which most were down-regulated at HT exposure; while the expression levels of *pyruvate kinase* (*PK*) increased at HT exposure. These genes regulated the conversion between hexoses, further promoting the accumulation of sugars. In addition, sucrose transporters (sucrose transporter (SUT) and sugars will eventually be exported transporters (SWEET)) changed significantly under different nitrogen-temperature treatments. The expression of *SUT* decreased at HTN1 vs CKN1, while it increased at HTN2 vs CKN2 and HTN3 vs CKN3; conversely, the expression of *SWEET* increased at HTN1 vs CKN1, while it decreased at HTN2 vs CKN2 and HTN3 vs CKN3, which ensured sugar transportation.

The organic acid metabolism of tomato is mainly the tricarboxylic acid cycle (TCA cycle), mainly involving citric acid metabolism and malic acid metabolism. The expression of *PEPC* increased under HTN1 vs CKN1 and HTN2 vs CKN2, while it decreased at HTN3 vs CKN3. In the comparison of the three groups, the expression of *CS* was down-regulated, while the expression of *IDH* was up-regulated, and the increase of HTN2 vs CKN2 and HTN3 vs CKN3 groups was higher than that of HTN1 vs CKN1. The expression of *cyt-ACO* was up-regulated in HTN2 vs CKN2, it was down-regulated in HTN1 vs CKN1 and HTN3 vs CKN3. The differential expression of these genes regulates the synthesis and degradation of citric acid. The expression of *PEPC* under HTN1 and HTN2 was increased, thus promoting the synthesis of malic acid. The down-regulation of *ME* expression inhibited the oxidative decarboxylation of malic acid to pyruvate. The expression levels of *MDH1* decreased at HT exposure. The expression of *MDH2* was up-regulated in HTN1 vs CKN1 and HTN3 vs CKN3, it was down-regulated in HTN2 vs CKN2. The differential expression of these genes regulates the synthesis and degradation of malic acid.

## 4 Discussion

Global warming is causing temperature changes on a scale unprecedented in the last 500 thousand years (Tiwari et al., 2020), and the frequency and intensity of global heat stress are steadily increasing (IPCC, 2021), with significant negative impacts on agricultural production. As an important agricultural country, China must face the challenges posed by global warming. Against this background, this study aims to simulate heat stress events through pot control experiments and determine the response of sugar and organic acid metabolism in tomato young fruits to heat stress. These results will help to develop reasonable and effective cultivation strategies for greenhouse tomatoes.

### 4.1 Appropriate N application improved the sucrose metabolism and transport under HT exposure

Soluble sugars, especially sucrose, fructose, and glucose, play a central role in metabolite signaling and response to external stresses in fruit structure and metabolism (Vijayakumar et al., 2021; Zhang et al., 2022). Studies have shown that HT can promote the accumulation of sugar content in fruits (Lokesha et al., 2019), thereby increasing the cell osmotic potential, reducing the cell water potential, and protecting the cell components from pressure-induced damage (Alsamir et al., 2021). In this study, the order of soluble sugar content among the six treatments was HTN1>HTN2>HTN3>CKN2>CKN3>CKN1 (**Figure 1D**). This result confirms that HT exposure significantly increases soluble sugar content in fruits, which is consistent with previous reports (Mesa et al., 2022). We also found that the soluble sugar content in tomato fruit was more sensitive to heat stress, indicating that HT environment was the main reason for the increase in soluble sugar content compared to N fertilization. Soluble sugar components responded differently to N levels under HT exposure. At the same N level, HT exposure significantly reduced sucrose content, whereas the sucrose content of HTN3 was significantly higher than that of CKN1 (**Figure 1A**), indicating that excessive nitrogen application under HT exposure may offset the negative effect of HT on sucrose. Under HT exposure, N1 had the highest glucose and fructose content, and with the increase of N application, the hexose content decreased (**Figures 1B, C**), suggesting that excessive N application under HT exposure may reduce the positive effect of HT on hexose. The highest soluble sugar content was observed under HTN1 treatment (**Figure 1D**), indicating that N1 was the optimal N application for tomato young fruits under HT exposure. In conclusion, under the interaction of HT and nitrogen fertilizer, the decrease in sucrose and the concomitant increase in glucose and fructose indicate that sucrose degradation dominates its synthesis as a strategy for tomato resistance to heat stress.

The content of soluble sugars and their components in fruits are strongly regulated by metabolism and transport. Invertases (AI and NI) irreversibly catalyze the hydrolysis of sucrose to glucose and fructose, which is crucial for the normal fruit development and response to biotic and abiotic stresses (Qin et al., 2016; Chen et al., 2021). In this study, AI activity of HTN2 and HTN3 was significantly increased, and NI activity was significantly increased at three N levels (**Figures 2A, C**). We also found a significant negative correlation between sucrose content and NI, while glucose content was significantly positively correlated with AI and NI (**Figure S2**), indicating that the increase in invertase activity regulated by N fertilizer under HT exposure was the reason for sucrose degradation. In addition, N2 and N3 treatments under HT exposure resulted in up-regulation of invertase genes, particularly the cell wall invertase *CWINV2* (*Solyc10g085650.2* and *Solyc10g085640.1*) (**Figure 8**), indicating that N fertilizer has a positive regulatory effect on *CWIN* expression under HT exposure. Up-regulation of *CWIN* expression accelerates sucrose degradation while reducing sucrose concentration in the fruit, promoting phloem unloading and generating sugar signals to regulate cell division, ultimately providing more energy and carbon scaffold for fruit development (Palmer et al., 2015; Li et al., 2016). In the early stages of fruit development, tomato fruits are a strong carbohydrate pool, and previous studies have suggested that SSc plays an important role in the formation of the pool because it can control the ability of tomato young fruits to unload sucrose (Alsamir et al., 2021). However, in this study, the results obtained have large variations. The SSc activity in response to HT was significantly increased at N2 and N3 levels but still significantly lower than AI (**Figures 2A, B**), and we could not find any significant correlation between it and sucrose content (**Figure S2**). Therefore, we speculated that N fertilizer regulates sucrose unloading ability of tomato young fruits by affecting AI activity and gene expression under HT stress, rather than SSc. SPS is a key enzyme that promotes sucrose biosynthesis in the cytoplasm and supports sugar exchange between the cytoplasm and vacuoles (Quinet et al., 2019). In this study, SPS activity and gene expression were up-regulated under HT exposure at three N levels (**Figures 2D**, **8**), suggesting that N fertilizer has a positive regulatory effect on SPS under HT exposure. We found a significant positive correlation among SPS activity, AI, and SSc, indicating that SPS can promote sucrose metabolism and contribute to increased sucrose unloading in fruits (Gao et al., 2022). N fertilizer regulates the interaction between sucrose and hexose in fruits by regulating the activity and gene expression of AI, NI, SS, and SPS under HT exposure, thereby controlling sugar content and composition.

Sucrose degrades to fructose and glucose, which are phosphorylated by *HK* and then enter glycolysis and the TCA cycle, providing energy for plant growth and development (Fernie et al., 2004; Zhu et al., 2013). *HK* activity is negatively correlated with hexose content (D'Aoust et al., 1999). In this study, *HK* expression was down-regulated under HT exposure at N2 and N3 (**Figure 8**), which may lead to a decrease in HK activity, limiting hexose metabolism and ultimately leading to hexose accumulation (**Figures 1B, C**). In particular, *HK2* (*Solyc06g066440.3*) was most down-regulated and played an important role in hexose accumulation at high temperatures. *PFK* expression was significantly downregulated in HTN1 and HTN3, suggesting that HTN1 inhibits the entry of glucose into glycolysis more strongly, leading to glucose accumulation (**Figure 1B**). These results clarify that N fertilizer increases the heat resistance of tomato fruit by regulating hexose content and hexose sensor activity.

In addition to structural enzymes involved in sucrose metabolism, sugar transporters (*SUTs* and *SWETs*) also play a key role in the soluble sugar profile (Schroeder et al., 2013). In this study, *SUT1* (Solyc11g017010.2) was up-regulated in HTN2 vs CKN2 (**Figure 8**), indicating that N2 treatment under HT could increase the concentration of sucrose in phloem sap. *SUT4* was significantly up-regulated under HT exposure, indicating that it played an important role in sugar accumulation of tomato fruit under HT exposure. The above results indicated that N fertilizer had different regulatory effects on *SUTs* under HT exposure. Most *SWEETs* were up-regulated under HT exposure (**Figure 8**), which promoted the sugar flux between intercellular spaces or between pulp cells and phloem, while *SWEET2* (*Solyc07g062120.4*) and *SWEET1* (*Solyc04g064610.3*) were down-regulated, indicating that these genes may act as exporters to extract sugar from the vacuolar membrane and reduce its concentration, similar to that reported previously (Yue et al., 2015). In summary, the changes in soluble sugar content and components in fruits were not regulated by a few/single genes, but rather by the joint efforts of multiple metabolic pathways.

### 4.2 Nitrogen application improved CA synthesis and TCA cycle, which contribute to organic acid accumulations in tomato young fruit

Sugar and organic acid metabolism are closely linked through the TCA cycle. Organic acids are the material basis of the TCA cycle and glycolysis and play an important role in fruit development (Li et al., 2021). In this study, under HT exposure, citric acid content significantly increased at N1 and N2 (**Figure 3B**), malic acid content increased at N1 (**Figure 3C**), while succinic acid, α-ketoglutaric acid, and tartaric acid content significantly decreased at the three N levels (**Figures 3D–F**). Therefore, HT at the N1 level significantly decreased while the N2 and N3 levels significantly increased the total acid content (**Figure 3A**).

The synthesis and conversion of citric acid are affected by the activities of CS, ACO, and IDH (Liao et al., 2022). Chen et al. (2012) found that citric acid content was positively correlated with CS and negatively correlated with ACO and IDH. However, Liao et al. (2019) found that the citric acid accumulation was regulated by ACO and not CS. In this study, we found a significant negative correlation between citric acid content and mit-ACO, whereas there was no significant correlation with CS activity (**Figure S2**). Because HT exposure significantly decreased CS activity at N3, we speculated that N fertilizer-induced changes in organic acids are normally triggered under excessive N conditions (Parisi et al., 2004). Consistent with the changes in enzymes, *CS* gene expression was downregulated under HT exposure. At the same N level, HT exposure significantly reduced the activities of mit-ACO and IDH, inhibiting citric acid degradation. This suggests that the accumulation of citric acid content in response to HT under N regulation is due to the weakened degradation of citric acid. IDH is the major rate-limiting enzyme in the TCA cycle (Wu et al., 2023). And it's also the key enzyme of C-N metabolism involved in N metabolism, glyoxylate cycle, and other biochemical metabolic pathways in plants (Anderson et al., 2000). Interestingly, although the activity of IDH decreased during HT exposure, the expression of *IDH* (*Solyc01g005560.3*, *Solyc11g011930.3*, *Solyc02g082860.3*) was significantly upregulated (**Figure 8**), which may be due the fact that tomato is forced to produce free oxygen under HT stress, which increases the content of free ammonium in cells involved in the glutamine synthase/glutamate synthase (GS/GOGAT) metabolic pathway. Up-regulation of *IDH* expression provided the necessary α-ketoglutarate for this metabolic pathway, which may reduce the toxicity of free ammonium to plants.

N application can affect the nitrogen metabolism of fruits. This study mainly investigated the regulation of nitrogen application on sugar and organic acid metabolism in tomato young fruits under HT exposure, with little involvement in its impact on fruit nitrogen metabolism. Therefore, the results of this study are similar to those of previous studies, but there are some differences, which may be related to this. The next research work will focus on the effect of nitrogen-temperature treatment on the nitrogen metabolism of tomato fruit.

## 5 Conclusions

Nitrogen fertilizer, HT, and their interactions had significant impact on the soluble sugar and organic acid content, composition, and metabolic enzyme activity of tomato young fruits. N fertilizer improved sugar metabolism under HT exposure by increasing the activity of AI, NI, SSc, and SPS, upregulating the expression of *CWINV2*, *HK2*, *SPS*, and *PK*, and increasing sucrose transport by upregulating *SUT1*, *SUT4*, and *SWEETs*, thereby improving the heat tolerance of tomato fruits. In addition, N fertilizer upregulated the gene expression of *PEPC* under HT exposure and downregulated the expression of *ACO*, *MDH*, and *ME*, thereby reducing the degradation of citric acid and malic acid, leading to the accumulation of citric acid and malic acid. The soluble sugar content and organic acid content were the highest under HTN1 treatment, while the soluble sugar content was the lowest under HTN3 treatment. Therefore, we concluded that N fertilizer increased the heat resistance of tomato young fruits and improved fruit quality mainly due to the upregulation of sucrose metabolism enzyme gene expression, and N1 was the optimal nitrogen application under HT exposure. These results will help to further develop reasonable and effective tomato fertilization strategies in the context of global warming.

## Data availability statement

The datasets presented in this study can be found in online repositories. The names of the repository/repositories and accession number(s) can be found below: NCBI Bioproject accession number: PRJNA948909.

## Author contributions

The YZhe and ZY designed the experiments, analyzed the data and wrote the manuscript. JL, YZha, and NJ performed the laboratory measurements. YZhe and ZY discussed the results and provide critical idea in greenhouse experiment. WK revised manuscript. All authors contributed to the article and approved the submitted version.

## Supplementary material

Supplementary Table 1 Detailed table of expression differences between different control groups.

Supplementary Table 2 Statistical table of GO classification between different control groups.

Supplementary Table 3 Statistical table for KEGG enrichment analysis between different control groups.
