# Supplementary material for: Transcriptome analysis of sugar and acid metabolism in young tomato fruits under high temperature and nitrogen fertilizer influence
Source: Front Plant Sci. 2023 Jul 19;14:1197553. doi: 10.3389/fpls.2023.1197553 (PMC10394703; doi:10.3389/fpls.2023.1197553)
Supplement: Supplementary file 1 [file DataSheet_1.docx]

Supplementary Material

Transcriptome analysis of sugar and acid metabolism in young tomato fruits under high temperature and nitrogen fertilizer influence

Yanjiao Zheng, Zaiqiang Yang^*^, Jing Luo, Yao Zhang, Yuhan Jiang, Nan Jiang, Wajid Ali Khattak

*** Correspondence:** Zaiqiang Yang: yzq@nuist.edu.cn

# Supplementary Figures and Tables

## Supplementary Figures


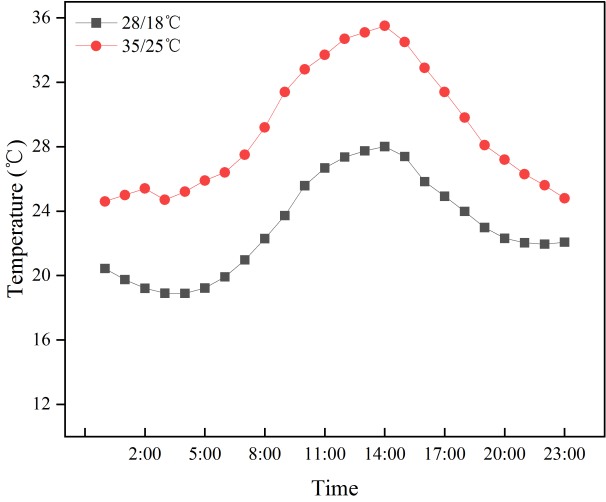


**Supplementary Figure 1.** The actual air temperature in CK (28/18℃) and heat stress (35/25℃) treatments in the artificial climate chambers.


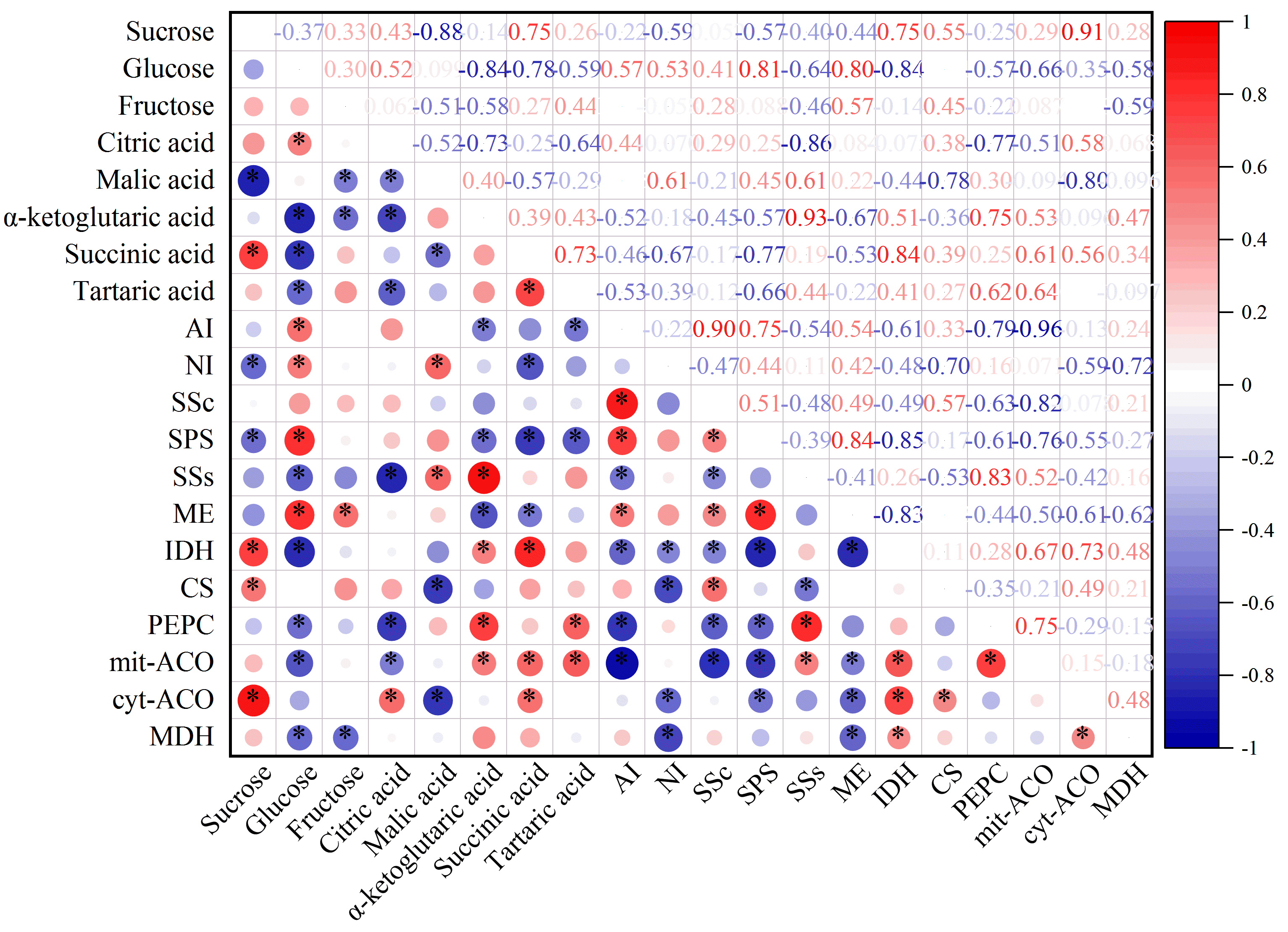


**Supplementary Figure 2.** The correlation between sugars, organic acids, and their metabolic enzyme activities. AI, acid invertase; NI, neutral invertase; SSc, sucrose synthase-cleavage; SSs, sucrose synthase-synthesis; SPS, sucrose phosphate synthase; PEPC, phosphoenolpyruvate carboxylase; MDH, malate dehydrogenase; ME, malic enzyme; CS, citrate synthase; cyt-ACO cytosolic aconitase; mit-ACO, mitochondrion aconitase; IDH, isocitrate dehydrogenase.  Positive values indicate positive correlation, while negative values indicate negative correlation. The darker the color, the stronger the correlation between the indicators. * represents the Pearson correlation coefficient *P* < 0.05.
